# Supplementary material for: Gastric Microbiota Gender Differences in Subjects with Healthy Stomachs and Autoimmune Atrophic Gastritis
Source: Microorganisms. 2023 Jul 29;11(8):1938. doi: 10.3390/microorganisms11081938 (PMC10456958; doi:10.3390/microorganisms11081938)

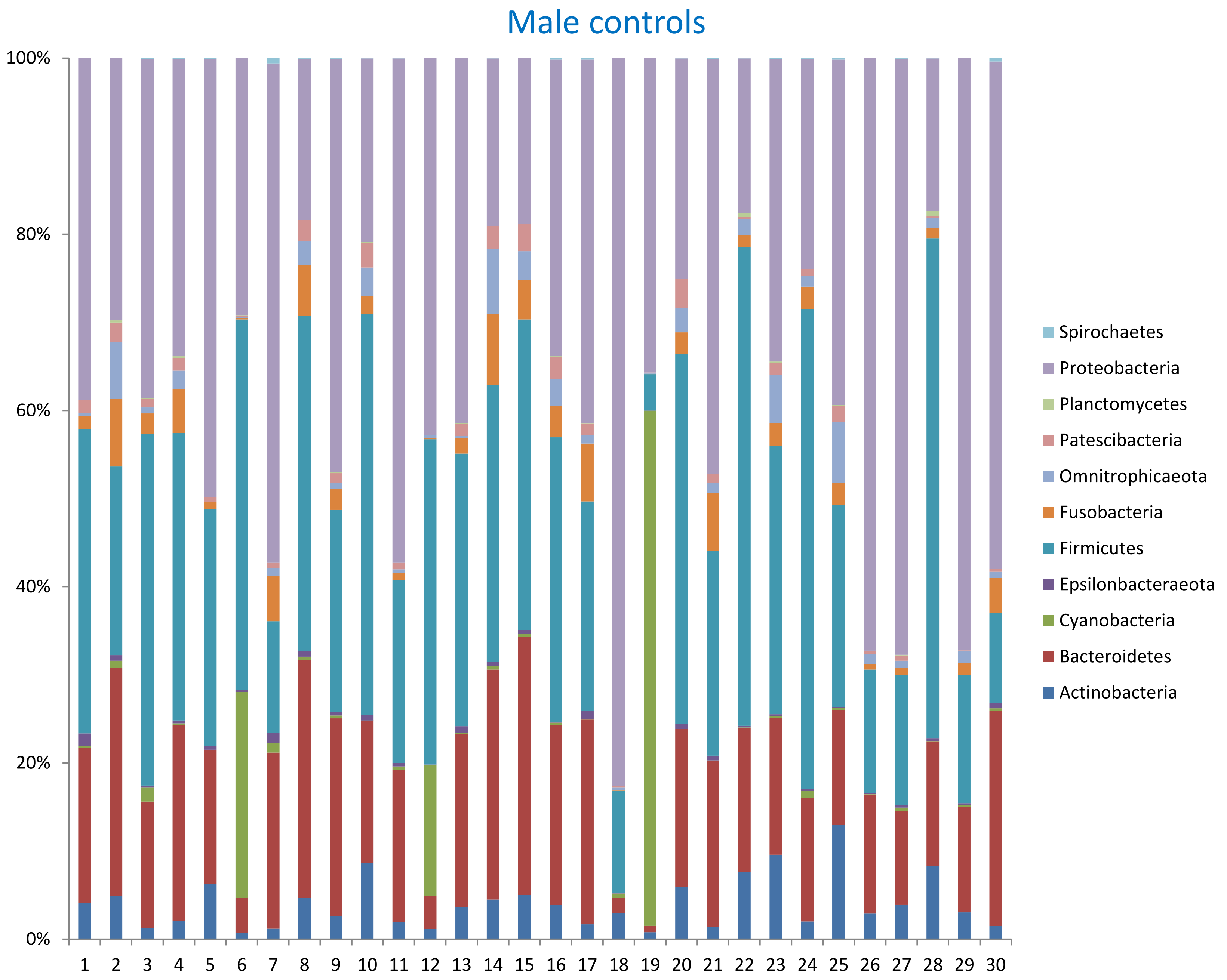

Male controls
100%
80%
60%
40%
20%
0%
1 2 3 4 5 6 7 8 9 10 11 12 13 14 15 16 17 18 19 20 21 22 23 24 25 26 27 28 29 30
Spirochaetes
Proteobacteria
Planctomycetes
Patescibacteria
Omnitrophicaeota
Fusobacteria
Firmicutes
Epsilonbacteraeota
Cyanobacteria
Bacteroidetes
Actinobacteria

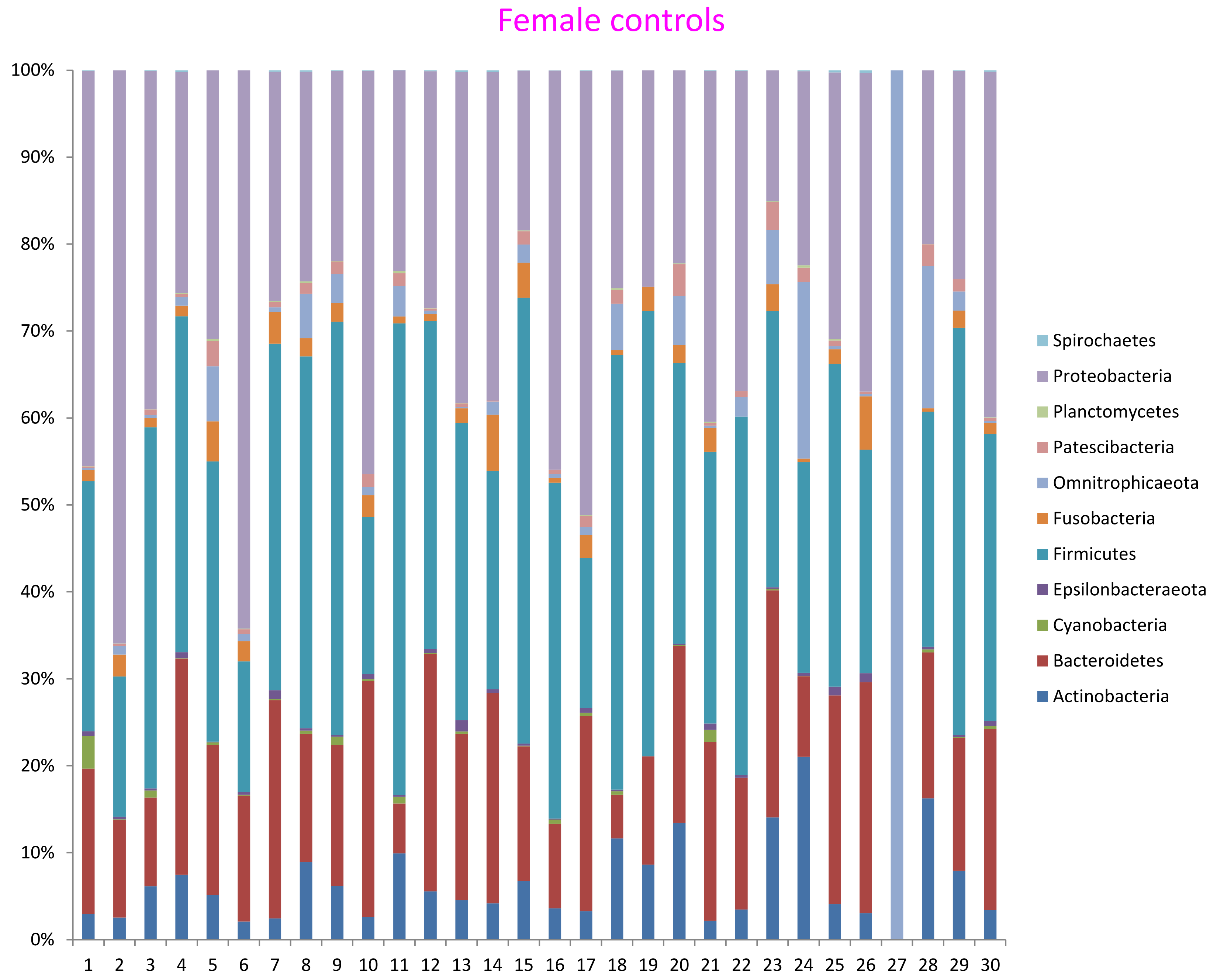
Female controls
100%
90%
80%
70%
60%
50%
40%
30%
20%
10%
0%
1 2 3 4 5 6 7 8 9 10 11 12 13 14 15 16 17 18 19 20 21 22 23 24 25 26 27 28 29 30
Spirochaetes
Proteobacteria
Planctomycetes
Patescibacteria
Omnitrophicaeota
Fusobacteria
Firmicutes
Epsilonbacteraeota
Cyanobacteria
Bacteroidetes
Actinobacteria

Male cases with autoimmune atrophic gastritis

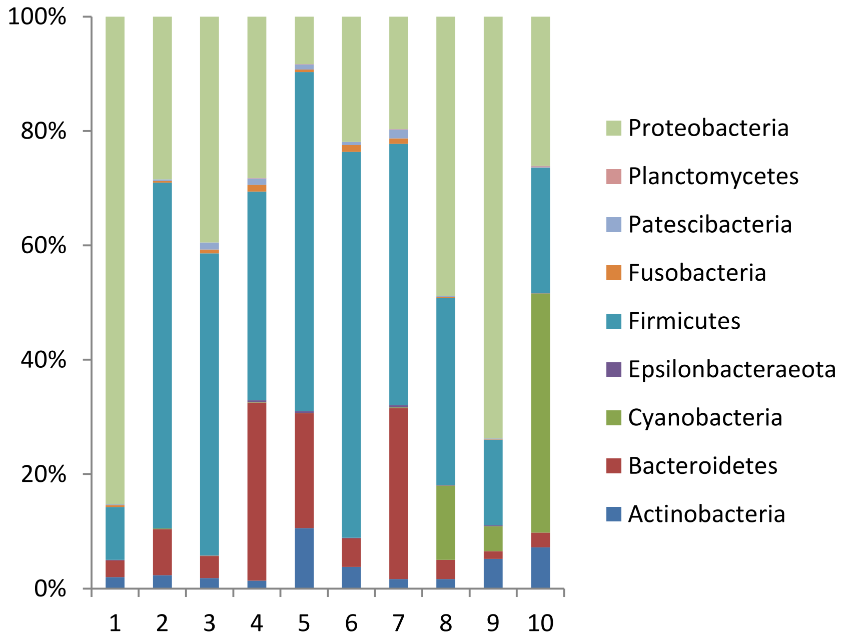

Female cases with autoimmune atrophic gastritis

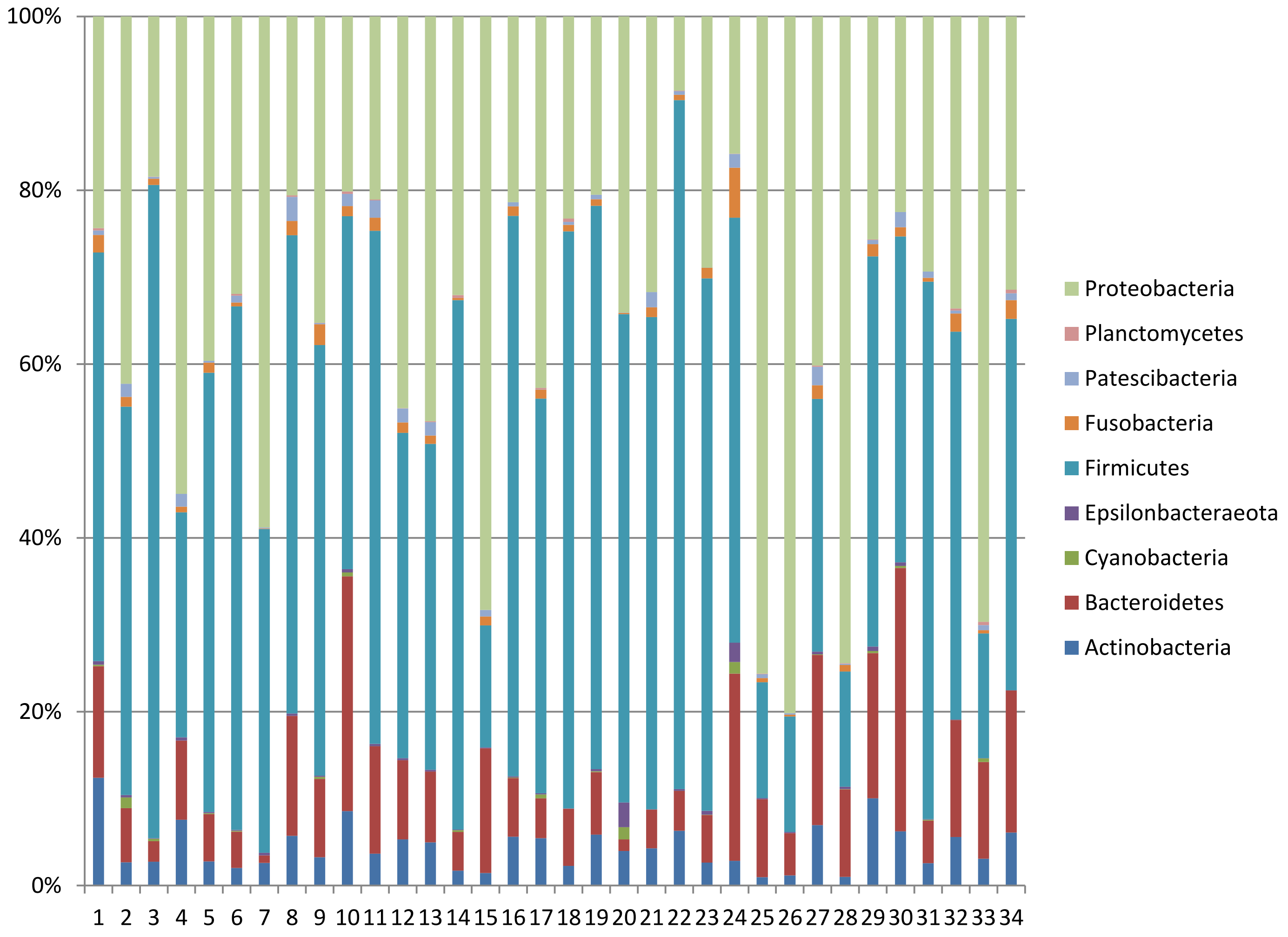

Supplement: Supplementary file 1 [file microorganisms-11-01938-s001.zip › microorganisms-2518012-supplementary.pdf]
